# Transient viscoelasticity study of tobacco mosaic virus/Ba^2+^ superlattice

**DOI:** 10.1186/1556-276X-9-300

**Published:** 2014-06-13

**Authors:** Haoran Wang, Xinnan Wang, Tao Li, Byeongdu Lee

**Affiliations:** 1 Department of Mechanical Engineering, North Dakota State University, Fargo ND 58108, USA; 2 X-ray Science Division, Advanced Photon Source of Argonne National Laboratory, 9700 S. Cass Avenue, Argonne IL 60439, USA

**Keywords:** Tobacco mosaic virus, Viscoelasticity, Atomic force microscopy, Nanoindentation

## Abstract

Recently, we reported a new method to synthesize the rod-like tobacco mosaic virus (TMV) superlattice. To explore its potentials in nanolattice templating and tissue scaffolding, this work focused the viscoelasticity of the superlattice with a novel transient method via atomic force microscopy (AFM). For measuring viscoelasticity, in contrast to previous methods that assessed the oscillating response, the method proposed in this work enabled us to determine the transient response (creep or relaxation) of micro/nanobiomaterials. The mathematical model and numerical process were elaborated to extract the viscoelastic properties from the indentation data. The adhesion between the AFM tip and the sample was included in the indentation model. Through the functional equation method, the elastic solution for the indentation model was extended to the viscoelastic solution so that the time dependent force *vs.* displacement relation could be attained. To simplify the solving of the differential equation, a standard solid model was modified to obtain the elastic and viscoelastic components of the sample. The viscoelastic responses with different mechanical stimuli and the dynamic properties were also investigated.

## Background

The recognition of tobacco mosaic virus (TMV) since the end of nineteenth century [1] has sparked innumerable research towards its potential applications in biomedicine [2,3] and biotemplates for novel nanomaterial syntheses [4,5]. A TMV is composed of a single-strand RNA that is coated with 2,130 protein molecules, forming a special tubular structure with a length of 300 nm, an inner diameter of 4 nm, and an outer diameter of 18 nm [6]. The TMVs observed under a microscope can reach several tens of microns in length due to its unique feature of head-to-tail self-assembly [7]. Practically useful properties of the TMVs include the ease of culture and broad range of thermal stability [8]. Biochemical studies have shown that the TMV mutant can function as extracellular matrix proteins, which guide the cell adhesion and spreading [8]. It has also been confirmed that stem cell differentiation can be enhanced by both native and chemically modified TMV through regulating the gene's expression [9-11]. Moreover, TMV can be electrospun with polyvinyl alcohol (PVA) into continuous TMV/PVA composite nanofiber to form a biodegradable nonwoven fibrous mat as an extracellular matrix mimetic [12].

**Figure 1:**
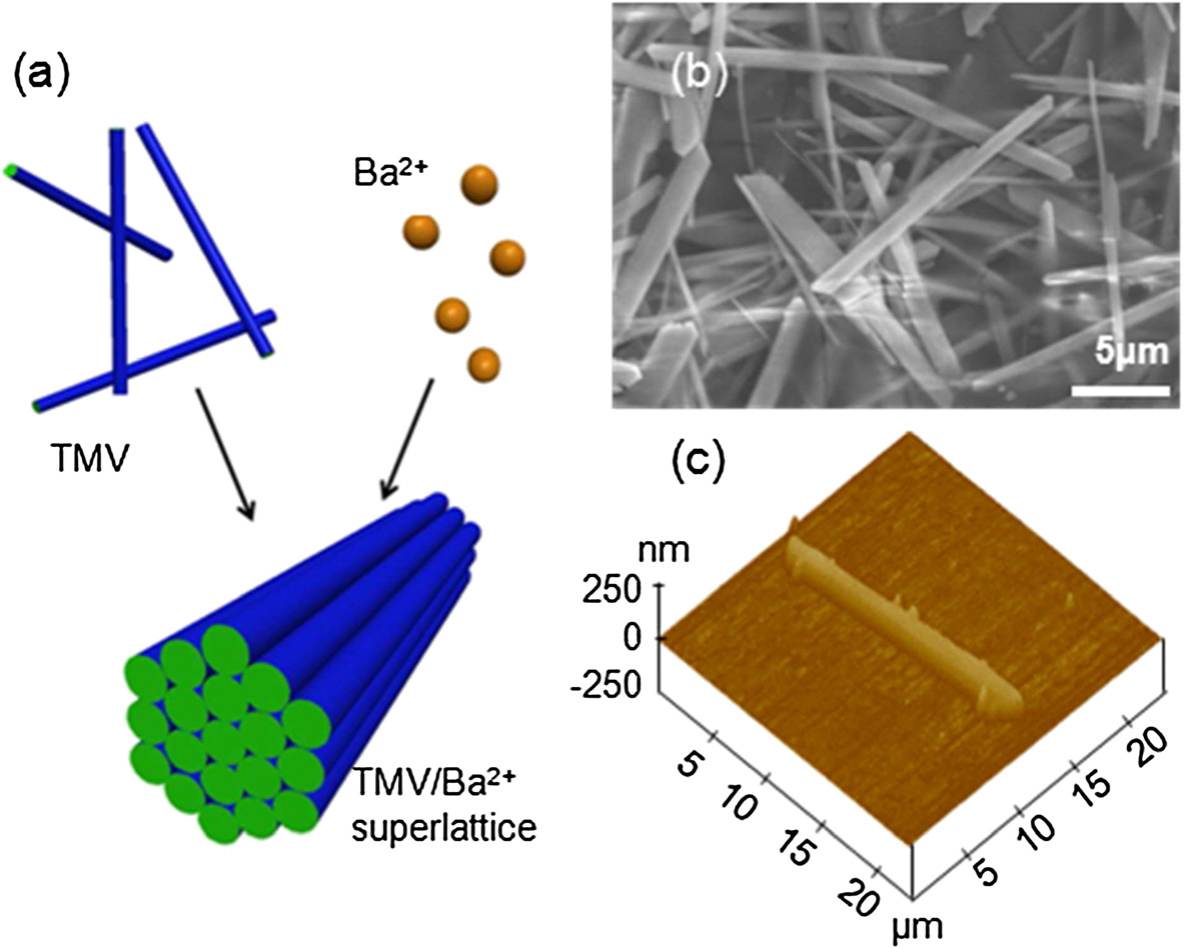
**Schematic, FESEM image, and AFM height image of TMV/Ba**^**2+**^**superlattice. (a)** Schematic of hexagonal organization of rod-like TMV/Ba^2+^ superlattice. **(b)** FESEM image of the TMV/Ba^2+^ superlattice. **(c)** AFM height image of a TMV/Ba^2+^ superlattice.

Very recently, we have reported that the newly synthesized hexagonally packed TMV/Ba^2+^ superlattice material can be formed in aqueous solution [13,14]. Figure 1 shows the schematic of the superlattice formation by hexagonal packing of TMVs, triggered by Ba ions, and the images observed from field emission scanning electron microscopy (FESEM) and atomic force microscopy (AFM). The sample we used for this experiment was tens of microns in length, 2 ~ 3 microns in width (from FESEM), and several hundred nanometers in height (from AFM height image). It is known that the superlattice exhibits physical and mechanical properties that differ significantly from its constituent materials [15-20]. The study on the viscoelastic properties of the TMV-derived nanostructured materials is still lacking despite the availability of the elastic property of the TMV and TMV-based nanotube composites [7]. The viscoelasticity of micro/nanobioarchitecture significantly affects the tissue regeneration [21] and repair [22], cell growth and aging [23], and human stem cell differentiation [24] as well as the appropriate biological functions of the membranes within a specific nanoenvironment [25]; in particular, the viscoelasticity of some viruses plays key roles in the capsid expansion for releasing nucleic acid and modifying protein cages for vaccine delivery purposes [26]. Specifically, for TMV superlattice, its nanotube structure makes it a perfect biotemplate for synthesizing nanolattices that have been confirmed to possess extraordinary mechanical features with ultralow density [27,28]. Considering the biochemical functions of the TMV, its superlattice is an excellent candidate for bone scaffolding where the time-dependent mechanical properties become determinant [29], and research on scaffolding materials remains a hotspot [30]. Apart from contributing to the application of TMV superlattice, this work also pioneered in the viscoelasticity study of virus and virus-based materials. By far, most literature on viral viscoelasticity has been focused on the dynamic properties of virus suspensions or solutions [31-34]. One of the rare viscoelasticity studies on individual virus particle is the qualitative characterization of the viscoelasticity of the cowpea chlorotic mottle virus [26] using quartz crystal microbalance with dissipation technique, which presents only the relative rigidity between two samples. To date, little literature is available on the quantitative study of the viscoelasticity of individual virus/virus-based particles. Considering the potential uses of TMV/Ba^2+^ superlattice, its viscoelastic properties and responses under different mechanical stimuli need to be investigated.

A number of techniques for measuring the viscoelasticity of macro-scale materials have been used. A comprehensive review of those methods can be found in the literature [35] that addresses the principles of viscoelasticity and experimental setup for time- and frequency-domain measurements. When the sample under investigation is in micro or even nanometer scale, however, the viscoelastic measurements become much more complicated. In dynamic methods, shear modulation spectroscopy [36] and magnetic bead manipulation [37] are two common methodologies to obtain the micro/nanoviscoelastic properties. To improve the measurement accuracy, efforts have been made to assess the viscoelasticity of micro/nanomaterials using contact-resonance AFM [38-41]. The adhesion between the AFM probe tip and sample, however, is usually neglected. Furthermore, in order for the dynamic method to obtain a sinusoidal stress response, the applied strain amplitude must be kept reasonably small to avoid chaotic stress response and transient changes in material properties [42]. In addition, the dynamic properties are frequency dependent, which is time consuming to map the viscoelasticity over a wide range of frequencies. An alternative way to measure the viscoelastic response of a material is the transient method. Transient indentation with an indenter was developed based on the functional equation methods [43], where the loading or traveling histories of the indenter need to be precisely programmed.

In this study, the viscoelastic properties of the TMV/Ba^2+^ superlattice were investigated using AFM-based nanoindentation. AFM has the precision in both force sensing and displacement sensing, although it lacks the programing capability in continuous control of force and displacement. To realize the transient indentation in AFM, we introduced a novel experimental method. Viscoelastic nanoindentation theories were then developed based on the functional equation method [44]. The adhesion between the AFM tip and the sample, which significantly affected the determination of the viscoelastic properties [45], was included in the indentation model [20]. The viscoelastic responses of the sample with respect to different mechanical stimuli, including stress relaxation and strain creep, were further studied. The transition from transient properties to dynamic properties was also addressed.

## Methods

The TMV/Ba^2+^ superlattice solution was obtained from the mixture of the TMV and BaCl_2_ solution (molar ratio of Ba^2+^/TMV = 9.2 × 10^4^:1) as stated in the reference [13]. It was further diluted with deionized water (volume ratio 1:1). A 10-μL drop of the diluted solution on a silicon wafer was spun at 800 rpm for 10 s to form a mono-layer dispersion of the sample. The sample was dried for 30 min under ambient conditions (40% R.H., 21°C) for AFM (Dimension 3100, Bruker, Santa Barbara, CA, USA) observation and subsequent indentation tests.

The sample was observed with FESEM and AFM. The indentation was performed using the AFM nanoindentation mode (AFM probe type: Tap150-G, NanoAndMore USA, Lady's Island, SC, USA). The geometry of the cantilever was precisely measured using FESEM (S-4700, Hitachi, Troy, MI, USA), with a length of 125 μm, width of 25 μm, and thickness of 2.1 μm. To accurately measure the tip radius, the tip was scanned on the standard AFM tip characterizer (SOCS/W2, Bruker) and the scanned data was curve fitted using PSI-Plot (Poly Software International, Orangetown, NY, USA). The tip radius calculated to be 12 nm. For a typical indentation test, the tip was pressed onto the top surface of the sample until a predefined force of ~100 nN. The cantilever end remained unchanged in position during the controlled delay time. A series of indentations of the same predefined indentation force and different delay times were performed to track the viscoelastic responses. A 10-min time interval of the two consecutive indentations was set for the sample to fully recover prior to the next indentation. The sample drift was minimized by turning off the light bulb in the AFM controller during scanning to keep the AFM chamber temperature constant and by shrinking the scan area gradually down to 1 nm × 1 nm on the top surface of the sample to rid the scanner piezo of the hysteresis effect.

### Mathematical formulation

Derived from the functional equation method and the standard solid model (shown in the ‘Appendix’), the differential equation governing the contact behavior of viscoelastic bodies can be obtained as

(1) $$\begin{array}{l} \left(\sum_{i=0}^{2}A_{i}\frac{\partial^{i}}{\partial t^{i}}\right)\left[F\left(t\right)+2\mathrm{\pi wR}\right]\\ \;=\frac{4\sqrt{R}}{3}\left(\sum_{i=0}^{2}B_{i}\frac{\partial^{i}}{\partial t^{i}}\right)\delta^{\frac{3}{2}}\left(t\right) \end{array}$$

where *F*(*t*) is the contact force history, *δ*(*t*) is the indentation depth history, *R* is the nominal radius of the two contact spheres, *w* is the adhesive energy density, *A*_*i*_ and *B*_*i*_ (*i* = 0, 1, 2) are the parameters determined by the mechanical properties of two contact bodies, and the calculation of all these parameters can be found in the ‘Appendix.’

**Figure 2:**
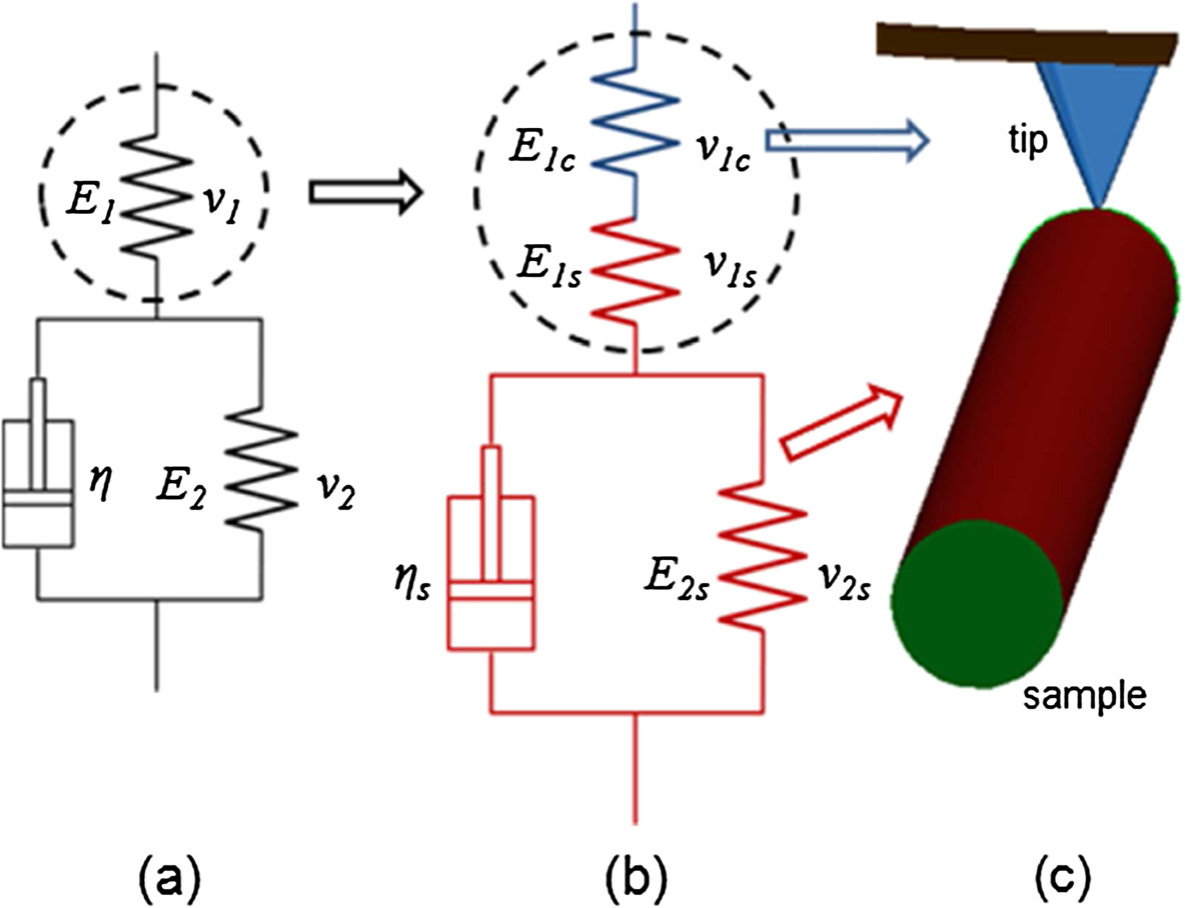
**Standard solid model and modified standard solid model. (a)** Schematic of the standard solid model for the TMV/Ba^2+^ superlattice sample. **(b/c)** Modified standard solid model with the cantilever denoted by the blue spring and the sample denoted by the red springs and dashpot.

**Figure 3:**
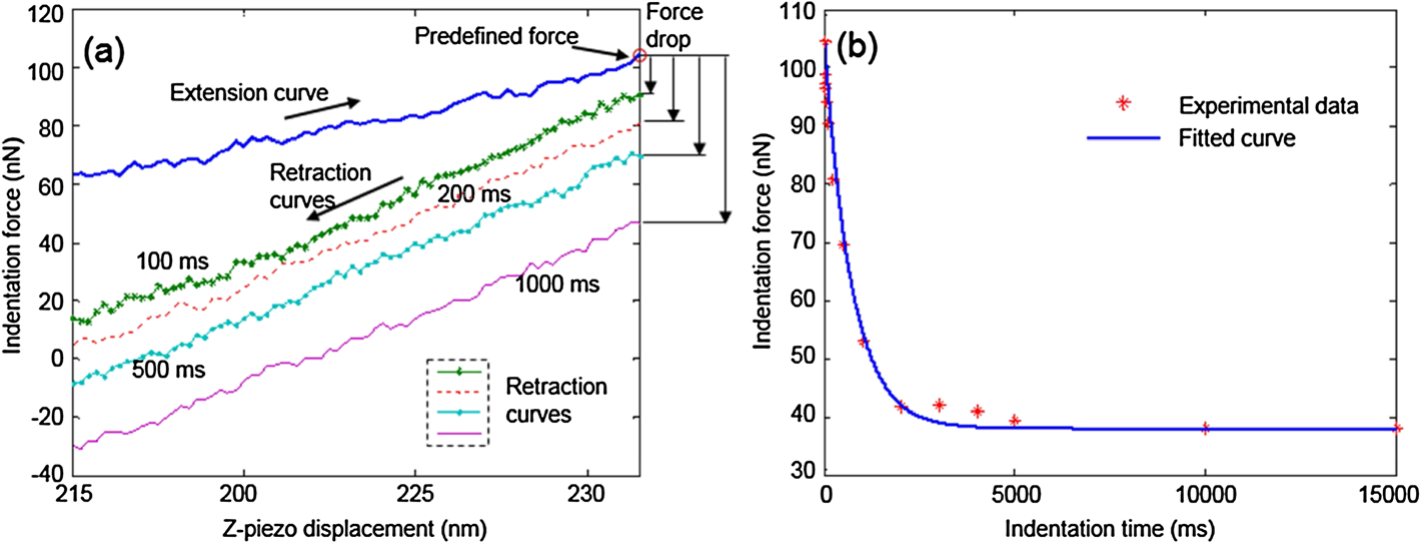
**Indentation force. (a)** Indentation force decrease with delay time set as 100 ms, 200 ms, 500 ms, and 1,000 ms, respectively. **(b)** Indentation force *vs.* time data from experiment measurement and fitted curve from the indentation equation.

The elastic moduli *E*_1_ and *E*_2_ and viscosity *η* in Figure 2 are implicitly included in the above differential equation. To determine *E*_1_, *E*_2_, and *η*, besides experimental data for *t* and *F*, the function of the force history *F*(*t*) is also required. The experimental data of *t* and *F* can be obtained as indicated in Figure 3. The force relaxation can be found in Figure 3a where the force decrease between the right ends of extension and retraction curves. By mapping the force decrease at different delay times as shown using the red asterisks in Figure 3b, the force relaxation curve can be obtained, which decreases from 104 to 40 nN. The function of *F*(*t*) can be obtained from Equation (1). Not only is Equation (1) applicable for the standard solid model in Figure 2(a) where it is derived from, but also it can be used for the modified standard solid model in Figure 2(b) where the elastic component of *E*_1_ is replaced by two elastic components in series. With this modification, the deflection of the cantilever can be incorporated into the deformation of the imaginary sample which is represented by the modified standard solid model where the elastic component of *E*_1c_ in Figure 2(b) denotes the cantilever and the rest components denote the TMV/Ba^2+^ superlattice.

**Figure 4:**
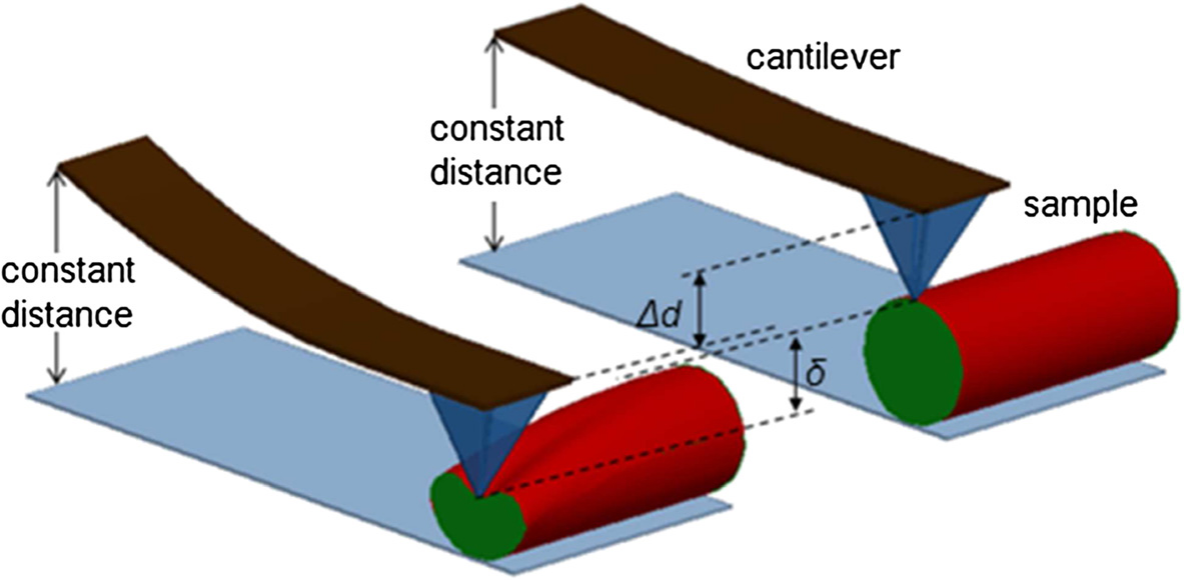
**Variation of cantilever deflection (∆*d*) and the sample deformation (*δ*) during indentation.** The sample is cut in half to show the deformation.

During each indentation, the vertical distance between the substrate and the end of the cantilever remains constant. Therefore, as the sample deformation or the indentation depth increases, the corresponding cantilever deflection ∆*d* or the normal indentation force decreases. During this process, the force on the system decreases while the sample deformation *δ* increases to compensate the decreased cantilever deflection. Therefore, the change of the cantilever deflection is equal to change of the sample deformation during indentation, as is shown in Figure 4. As such, *δ* in Equation (1) represents the relative approach between the cantilever end and the substrate, which incorporates the deformation of both the sample and the cantilever.

To be clearer, *δ* is substituted by D which represents the combined deformation. The relative approach, D, can be written as

(2) $$D\left(t\right)=D_{0}H\left(t\right)$$

where *H*(*t*) is the Heaviside unit step function and D_0_ is the relative approach between the substrate and the end of the cantilever.

Thus, Equation (1) can be rewritten as

(3) $$\left(\sum_{i=0}^{2}A_{i}\frac{\partial^{i}}{\partial t^{i}}\right)\left(F\left(t\right)+2\mathrm{\pi wR}\right)=\left(4\sqrt{R}/3\right)D_{0}^{\frac{3}{2}}\left(\sum_{i=0}^{2}B_{i}\frac{\partial^{i}}{\partial t^{i}}\right)H\left(t\right)$$

Applying Laplace transform, it yields

(4) $$\begin{array}{l} \left(A_{0}+A_{1}s+A_{2}s^{2}\right)\left(\hat{F}\left(s\right)+\frac{2\mathrm{\pi wR}}{s}\right)\\ \;=\left(4\sqrt{R}/3\right)D_{0}^{\frac{3}{2}}\left(B_{0}+B_{1}s+B_{2}s^{2}\right)\frac{1}{s} \end{array}$$

where a function with ‘^∧^’ denotes Laplace-transformed function in *s* domain.

Performing inverse Laplace transform, the viscoelastic equation of AFM-based indentation becomes

(5) $$F\left(t\right)=\frac{4\sqrt{D_{0}^{3}R}}{3}\left(A_{r}e^{-\alpha \;t}+B_{r}e^{-\beta \;t}+C_{r}\right)-2\mathrm{\pi wR}$$

where

$$\begin{array}{ll} A_{r} & =\frac{G_{1}^{2}}{G_{1}+G_{2}},B_{r}\\ & =\frac{27G_{1}^{2}K_{1}^{2}}{\left(3K_{1}+4G_{1}\right)\left(3K_{1}G_{1}+3K_{1}G_{2}+4G_{1}G_{2}\right)} \end{array}$$

$$\begin{array}{l} C_{r}=\frac{4G_{1}G_{2}}{G_{1}+G_{2}}\left(1-\frac{3G_{1}G_{2}}{3K_{1}G_{1}+3K_{1}G_{2}+4G_{1}G_{2}}\right),\\ \alpha =\frac{G_{1}+G_{2}}{\eta },\;\beta =\frac{G_{2}}{\eta }+\frac{3K_{1}G_{1}}{\eta \left(3K_{1}+4G_{1}\right)} \end{array}$$

### Solution to AFM-based indentation equation

It is observed from Figure 3 that the initial indentation force at *t* = 0 was measured to be 104.21 nN, then the force started to decrease and then remained constant at 38 nN after ~5,000 ms. The force decrease shown as red asterisks in Figure 3b fits qualatitatively well with the exponential function of Equation (5). *E*_1_, *E*_2_, and *η*, corresponding to the mechanical property parameters in Figure 2(a), can then be determined by fitting Equation (5) with the experimental data.

From the indentation data, D_0_ is obtained to be 78.457 nm. The pull-off force, 2*πwR*, calculated by averaging the pull-off forces of multiple indentations on the sample, is 16 nN. In comparison with the radius of the AFM tip, the surface of the sample can be treated as a flat plane. Hence, the nominal radius *R = R*_tip_ *=* 12 nm.

By invoking the force values at *t =* 0, *t* = ∞, and any intermediate point into Equation (5), the elasticity and viscosity components can be determined to be *E*_1_ *=* 32.0 MPa, *E*_2_ *=* 21.3 MPa, and *η =* 12.4 GPa ms. The coefficient of determination *R*^2^ of the viscoelastic equation and the experimental data is ~0.9639.

Since the stress relaxation process is achieved by modeling a combination of the cantilever and the sample, the viscoelasticity of the sample can be obtained by subtracting the component of the cantilever from the results. The cantilever, acting as a spring, is in series with the sample, represented by a standard solid model. The schematic of the series organization is shown in Figure 2(b). Thus the component of *E*_1_ comprises of *E*_1*s*_ representing the elastic part from the sample and *E*_1c_ representing the elastic part from the cantilever. To clarify the sources of the components in the modified standard solid model, *E*_2_, *v*_2_, and *η* in Figure 2(a) are now respectively denoted by *E*_2*s*_, *v*_2*s*_, and *η*_*s*_ in Figure 2(b), where the subscript ‘s’ denotes the sample.

At the onset of indentation, only the spring with elastic modulus of *E*_1_ takes the instantaneous step load; therefore, the elastic modulus of *E*_1*s*_ can be determined from the experimental data of zero-duration indentation. Applying the DMT model [46] with the force-displacement relationship of the cantilever,

(6) $$F=k\delta_{\mathrm{cantilever}}$$

we can obtain the elastic equation of AFM-based indentation

(7) $$\delta =\frac{F}{k}+{\left(\frac{F+2\mathrm{\pi wR}}{E^{*}\sqrt{R}}\right)}^{\frac{2}{3}}$$

where *k* is the spring constant of the cantilever, which is 5 nN/nm based on Sader's method [47] to calibrate *k*, *δ*_cantilever_ is the cantilever deflection, and *δ* is recorded directly as the Z-piezo displacement by AFM.

**Figure 5:**
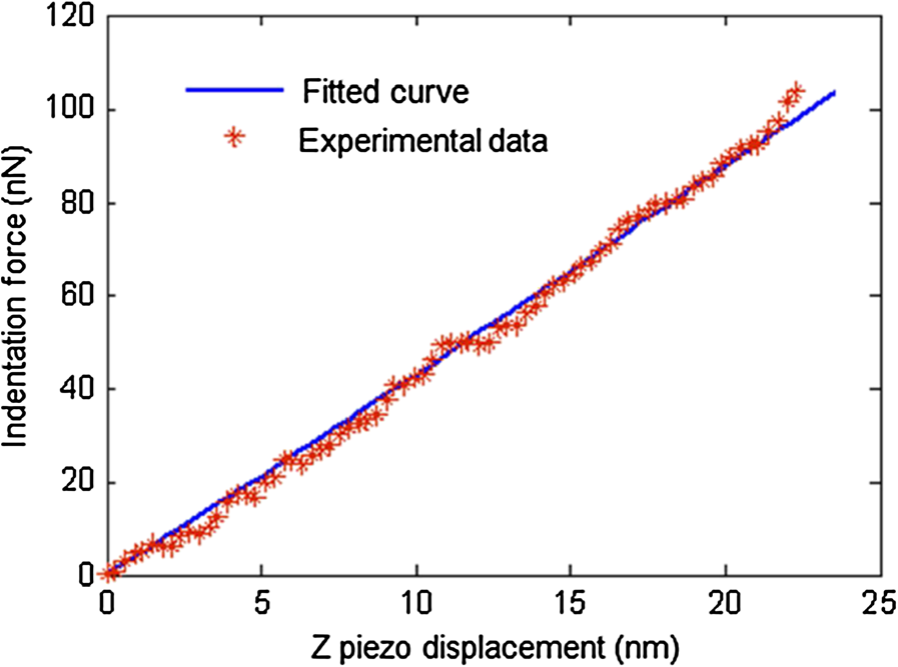
Indentation force data as a function of Z-piezo displacement, a comparison of experimental measurement and fitted results.

The elastic modulus of *E*_1*s*_ can be calculated by fitting the DMT-model-based indentation equation with experimental data as shown in Figure 5. For simplicity, modification was done to the indentation equation and the experimental data, whose details can be found in reference [20]. The fitted elastic modulus of *E*_1*s*_ is ~2.14 GPa with a coefficient of determination of 0.9948.

## Results and discussion

Based on the solution obtained, the viscoelastic equation of AFM-based indentation for TMV/Ba^2+^ superlattice is written as

(8) $$F\left(t\right)=3.2098\left(0.007e^{-\frac{0.0193\;t}{12.4}}+0.0136e^{-\frac{0.0163\;t}{12.4}}+0.0168\right)-16$$

The force decrease curve is shown in Figure 3b with the experimental data.

Specifically, for the TMV/Ba^2+^ superlattice whose viscoelastic behavior is simulated by a standard solid model, the differential equation governs its stress-strain behavior and becomes

(9) $$\dot{\sigma }+\frac{E_{2s}}{\eta_{s}}\sigma =\frac{E_{1s}E_{2s}}{\eta_{s}}\epsilon +\left(E_{1s}+E_{2s}\right)\dot{\epsilon }$$

where *E*_1*s*_ *=* 3 GPa, *E*_2*s*_ = 21.3 MPa, and *η*_*s*_ *=* 12.4GPa ms.

In the standard solid model, the initial experimental data point is determined by the instantaneous elastic modulus *E*_1*s*_. For the indentation that is held for over 5,000 ms, the indentation force becomes steady at ~38 nN, when the force exerts on the two springs in series. In contrast to *E*_1*s*_, *E*_2*s*_ is much smaller, as can be seen from the significant force decrease of from ~104 to ~38 nN. The tip traveled down 13.2 nm from the beginning of indentation. It is noted that for our indentation test, the ratio of the maximum indentation depth to the sample diameter is less than 10% [48,49]; the substrate effect to the elastic modulus calculation is neglected.

From the determined viscoelastic model, the mechanical response of the superlattice under a variety of mechanical loads can be predicted. Several simulation results were included as follows.

When the TMV/Ba^2+^ superlattice sample undergoes a uniformly constant tensile/compressive strain, the stress relaxation can be obtained from the standard solid model as below

(10) $$\sigma \left(t\right)=\epsilon_{0}\left(E_{1s}+E_{2s}e^{-E_{2s}t/\eta }\right)$$

where *ϵ*_0_ is the constantly applied strain.

When the sample undergoes a uniformly constant tensile/compressive stress, the strain creep can then be obtained as

(11) $$\epsilon \left(t\right)=\sigma_{0}\left[\frac{1}{E_{1s}}+\left(\frac{1}{E_{1s}+E_{2s}}-\frac{1}{E}\right)e^{-E_{1s}E_{2s}t/\eta_{s}\left(E_{1s}+E_{2s}\right)}\right]$$

where *σ*_0_ is the constantly applied stress.

**Figure 6:**
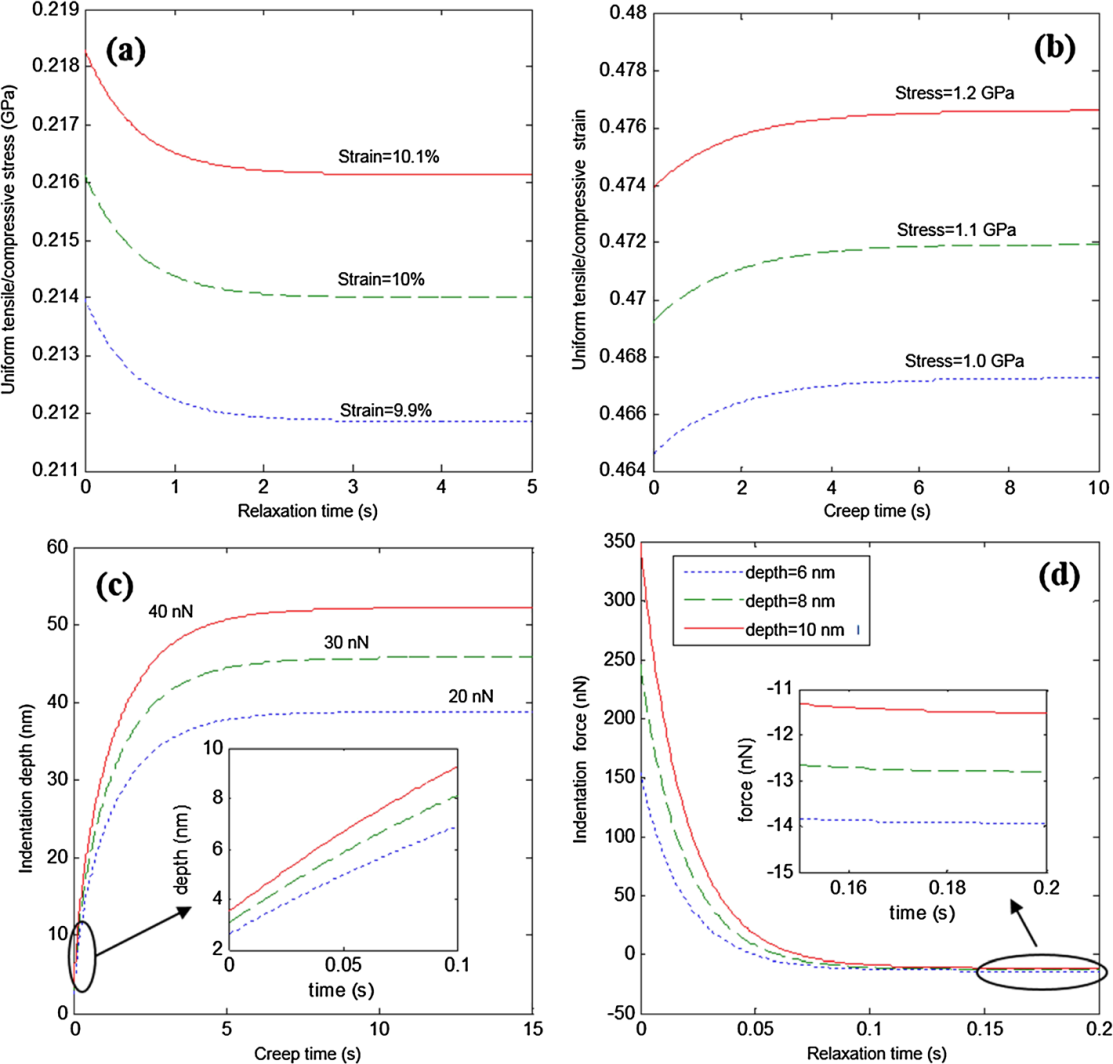
**Stress relaxation, strain creep, and indention depth creep and force relaxation. (a)** Stress relaxation of TMV/Ba^2+^ superlattice under uniform tensile/compressive strains. **(b)** strain creep under uniform tensile/compressive stresses. **(c)** Indentation depth creep with a rigid spherical indenter (*R* = 12 nm) under constant forces. **(d)** Indentation force relaxation with a rigid spherical indenter (*R* = 12 nm) under constant indentation depths.

**Figure 7:**
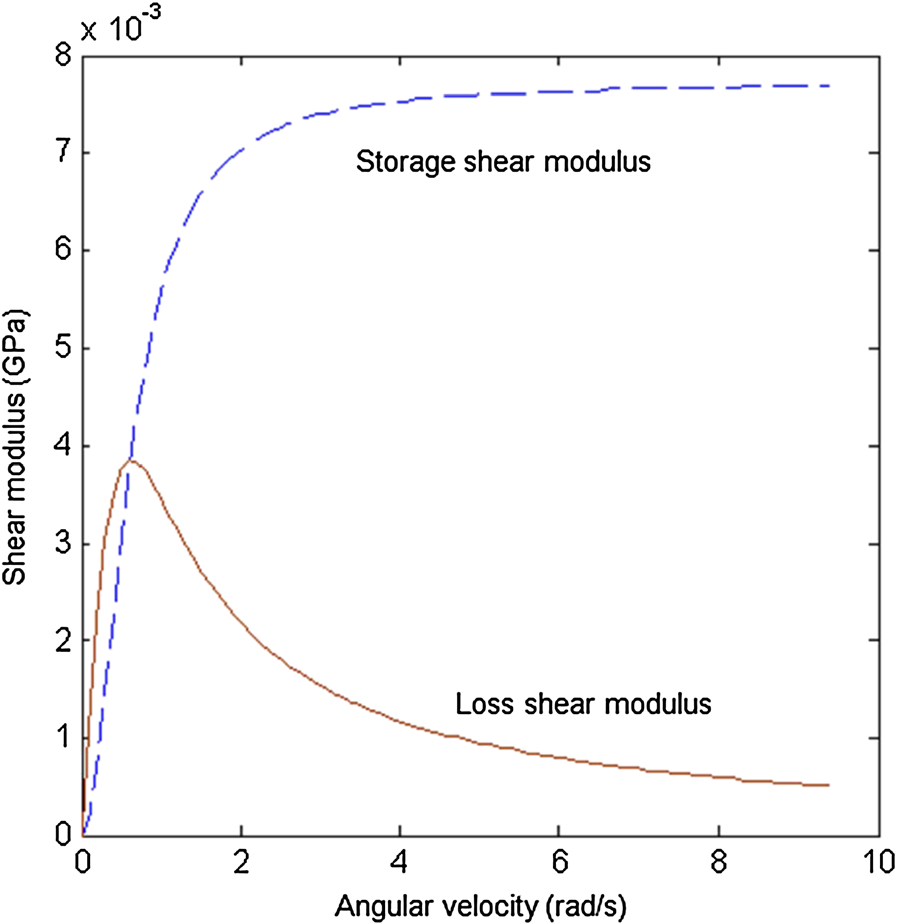
**Storage and loss shear moduli *vs. *** **angular velocity.**

The stress relaxation *vs.* applied strains and the strain creep *vs.* applied stresses are shown in Figure 6a,b, respectively. In Figure 6a, the stress reduces to a steady state after ~2 s when the applied strain is ~10%. In Figure 7b, strain increases to a steady value after ~5 s when the applied stress is ~ 1 GPa.

When the sample is indented with a spherical indenter, the indentation depth history can be analytically obtained when a step force is applied. Similar to the procedures above where the force history of Equation (5) is obtained, a step force function is used as input, and the creep indentation depth history function can be derived as

(12) $$\begin{array}{ll} d\left(t\right) & =\left\{\frac{3\left(F_{0}+2\mathrm{\pi wR}\right)}{4\sqrt{R}}\frac{G_{1s}+G_{2s}}{2G_{1s}\left(1+6K_{1s}\right)}\right)\\ & \;{\left(\left[A'+\frac{B'}{\eta }e^{\frac{-G_{2s}t}{\eta_{s}}}+\frac{C'\left(G_{1s}+G_{2s}\right)}{\eta_{s}}e^{\frac{-\left(G_{1s}+G_{2s}\right)t}{\eta_{s}}}\right]\;\right\}}^{\frac{2}{3}} \end{array}$$

where F_0_ is the step force, $A'=\frac{4G_{1s}}{G_{1s}+G_{2s}}+\frac{3K_{1s}}{G_{2s}}$

$$B'=\frac{4G_{1s}G_{2s}^{2}\eta_{s}\left(1-G_{1s}-G_{2s}\right)-3K_{1s}\eta_{s}G_{1s}G_{2s}\left(G_{1s}+2G_{2s}+1\right)+3K_{1s}\eta_{s}\left(G_{2s}^{2}-G_{1s}^{2}-G_{2s}^{3}\right)}{G_{2s}\left(G_{1s}+G_{2s}\right)\left(G_{1s}+2G_{2s}\right)}$$

$$C'=\frac{4G_{1s}G_{2s}\eta_{s}-3K_{1s}\eta_{s}G_{1s}-3K_{1s}\eta_{s}G_{2s}^{2}}{{\left(G_{1s}+G_{2s}\right)}^{2}\left(G_{1s}+2G_{2s}\right)}-\frac{4G_{1s}G_{2s}\eta_{s}}{\left(G_{1s}+2G_{2s}\right)\left(G_{1s}+G_{2s}\right)}-\frac{3K_{1s}\eta_{s}G_{1s}}{{\left(G_{1s}+G_{2s}\right)}^{2}}+\frac{3K_{1s}\eta_{s}\left(G_{2s}-G_{1s}\right)}{G_{2s}\left(G_{1s}+G_{2s}\right)\left(G_{1s}+2G_{2s}\right)}+\frac{3K_{1s}\eta_{s}G_{1s}}{G_{2s}{\left(G_{1s}+G_{2s}\right)}^{2}}$$

$$G_{1s}=\frac{E_{1s}}{2\left(1+v_{1s}\right)},K_{1s}=\frac{E_{1s}}{3\left(1-2v_{1s}\right)}.$$

The indentation force history has been obtained in Equation (5), where the elastic shear modulus *G*_1_ as a combined elastic response of two springs shown in Figure 2(b) should be replaced by *G*_1*s*_ of one spring only. Then, the simulated curves for the two situations can be found in Figures 6c,d. It is concluded that the creep depth variation under different forces gets larger through creep while the indentation force variation under different depths gets smaller through relaxation. Particularly, in Figure 6d, the force finally decreases to negative values, which represent attractive forces. The attraction cannot be found when *G*_1*s*_ and *G*_2*s*_ are very small. This phenomenon can be interpreted by the conformability of materials determined by the elastic modulus. When *G*_1*s*_ and *G*_2*s*_ get smaller, the materials are more conformable. Accordingly, in the final equilibrium state, the materials around the indenter tend to be more deformable to enclose the spherical indenter. This will result in a smaller attraction.

In addition, the example of shear dynamic experiment is simulated to obtain the storage and loss moduli of TMV/Ba^2+^ superlattice. The storage and loss shear moduli are calculated by [42]

(13) $$G'\left(\omega \right)=\omega \int_{0}^{\infty }G_{s}\left(t\right)\sin\mathrm{\omega tdt}$$

(14) $$G"\left(\omega \right)=\omega \int_{0}^{\infty }G_{s}\left(t\right)\cos\mathrm{\omega tdt}$$

where *G′* and *G″* are storage and loss moduli, respectively, *ω* is the angular velocity which is related to the frequency of the dynamic system, and $G_{s}\left(t\right)=G_{1s}+G_{2s}e^{-G_{2s}t/\eta }$ is the shear stress relaxation modulus, determined by the ratio of shear stress and constant shear strain.

Based on the relation between the transient and dynamic viscoelastic parameters in Equations (13) and (14), the storage and loss shear moduli are finally determined to be

(15) $$G'\left(\omega \right)=\frac{\omega^{2}G_{2s}\eta_{s}^{2}}{G_{2s}^{2}+\omega^{2}\eta_{s}^{2}}$$

(16) $$G\prime \prime \left(\omega \right)=\frac{G_{2s}^{2}\omega \eta_{s}}{G_{2s}^{2}+\omega^{2}\eta_{s}^{2}}$$

where *G*_2*s*_ = *E*_2*s*_ / 2(1 + *v*_2*s*_).

Figure 7 shows the curves of storage and loss shear moduli *vs.* the angular velocity. The storage shear modulus, *G′*, increases with the increase of angular velocity, while the increasing rate of *G′* decreases and the angular velocity of ~2 rad/s is where the increasing rate changes most drastically. However, the loss shear modulus, *G″*, first increases and then decreases reaching the maximum value, ~3.9 MPa, at the angular velocity of ~0.7 rad/s. The storage and loss moduli in other cases as uniform tensile, compressive, and indentation experiments can also be obtained.

## Conclusions

This paper presented a novel method to characterize the viscoelasticity of TMV/Ba^2+^ superlattice with the AFM-based transient indentation. In comparison with previous AFM-based dynamic methods for viscoelasticity measurement, the proposed experimental protocol is able to extract the viscosity and elasticity of the sample. Furthermore, the adhesion effect between the AFM tip and the sample was included in the indentation model. The elastic moduli and viscosity of TMV superlattice were determined to be *E*_1*s*_ *=* 2.14 GPa, *E*_2*s*_ = 21.3 MPa, and *η*_*s*_ *=* 12.4 GPa∙ms. From the characterized viscoelastic parameters, it can be concluded that the TMV/Ba^2+^ superlattice was quite rigid at the initial contact and then experienced a large deformation under a constant pressure. Finally, the simulation of the mechanical behavior of TMV/Ba^2+^ superlattice under various loading cases, including uniform tension/compression and nanoindentation, were conducted to predict the mechanical response of sample under different loadings. The storage and loss shear moduli were also demonstrated to extend the applicability of the proposed method. With the characterized viscoelastic properties of TMV superlattice, we are now able to predict the process of tissue regeneration around the superlattice where the time-dependent mechanical properties of scaffold interact with the growth of tissue.

## Appendix

### Modeling of adhesive contact of viscoelastic bodies

The functional equation method was employed to develop a contact mechanics model for indenting a viscoelastic material with adhesion. A modified standard solid model was used to extract the viscous and elastic parameters of the sample.

Several adhesive contact models are available, such as Johnson-Kendall-Roberts (JKR) model [50], Derjaguin-Muller-Toporov (DMT) model [46], *etc*. [51-53]. Detailed comparisons can be found in reference [54]. As the DMT model results in a simpler differential equation, it was used in this study for the simulation to solve the indentation on an elastic body with adhesion.

**Figure 8:**
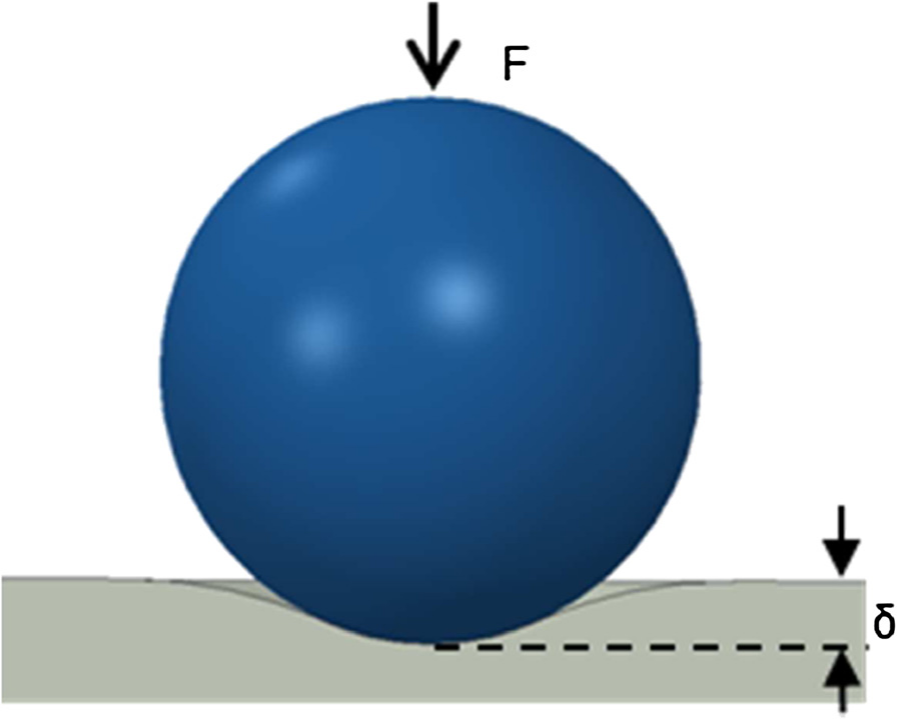
Schematic of contact between a rigid sphere and a flat surface (cross-section view).

For the DMT model [46], the relation between the indentation force *F* and relative approach *δ*, shown in Figure 8, can be expressed as

(A.1) $$F+2\mathrm{\pi wR}=\delta^{\frac{3}{2}}E^{*}\sqrt{R}$$

where *R* is the nominal radius of the two contact spheres of *R*_1_ and *R*_2_, given by *R* = *R*_1_*R*_2_/(*R*_1_ + *R*_2_); the adhesive energy density *w* is obtained from the pull-off force *F*_*c*_, where *F*_*c*_ = 3*πwR*/2; and the reduced elastic modulus E^*^ is obtained from the elastic modulus *E*_*s*_ and Poisson's ratio *ν*_*s*_ of the sample by $E^{*}=4E_{s}/\left[3\left(1-v_{s}^{2}\right)\right]$ with the assumption that the elastic modulus of the tip is much larger than that of the sample.

In Equation (A.1), E^*^, which governs the contact deformation behavior, is decided by the sample's mechanical properties. In the functional equation method [43], E^*^ needs to be replaced by its equivalence in the viscoelastic system, so that the contact deformation behavior can be governed by the viscoelastic properties. To achieve it, the elastic/viscoelastic constitutive equations are needed.

As a premise of the functional equation method, quasi-static condition is assumed so that the inertial forces of deformation can be neglected [43,44]. The general constitutive equations for a linear viscoelastic/elastic system in Cartesian coordinate configuration can be written as

(A.2) $$P^{d}s_{\mathrm{ij}}=Q^{d}e_{\mathrm{ij}}$$

(A.3) $$P^{m}\sigma_{\mathrm{kk}}=Q^{m}\epsilon_{\mathrm{kk}}$$

where *s*_*ij*_, *e*_*ij*_, *σ*_kk_, and *ϵ*_*kk*_ are the deviatoric stress, strain, mean stress, and strain, respectively. The linear operators *P*^*d*^, *Q*^*d*^, *P*^*m*^, and *Q*^*m*^ can be expressed in the form of

(A.4a) $$P^{d}=\sum_{i=0}^{N_{1}}p_{i}^{d}\frac{\partial^{i}}{\partial t^{i}},\;Q^{d}=\sum_{i=0}^{N_{2}}q_{i}^{d}\frac{\partial^{i}}{\partial t^{i}}$$

(A.4b) $$P^{m}=\sum_{i=0}^{N_{3}}p_{i}^{m}\frac{\partial^{i}}{\partial t^{i}},\;Q^{m}=\sum_{i=0}^{N_{4}}q_{i}^{m}\frac{\partial^{i}}{\partial t^{i}}$$

where *i* (*i =* 0, 1, 2,…) is determined by the viscoelastic model to be selected, *t* is time, and $p_{i}^{d}$, $q_{i}^{d}$, $p_{i}^{m}$, and $q_{i}^{m}$ are the components related to the materials property constants, such as elastic modulus and Poisson's ratio *etc*.

For a pure elastic system, the four linear operators are reduced to

(A.5) $$P^{d}=p_{0}^{d},\;Q^{d}=q_{0}^{d},\;P^{m}=p_{0}^{m},\;Q^{m}=q_{0}^{m}$$

which, according to the elastic stress-strain relations, are correlated as

(A.6) $$\frac{q_{0}^{d}}{p_{0}^{d}}=2G=\frac{Q^{d}}{P^{d}},\frac{q_{0}^{m}}{p_{0}^{m}}=3K=\frac{Q^{m}}{P^{m}}$$

where *G* and *K* are the shear modulus and bulk modulus, respectively.

Combining Equation (A.6) with

(A.7) $$G=\frac{E}{2\left(1+v\right)},K=\frac{E}{3\left(1-2v\right)}$$

the reduced elastic modulus can be expressed by the elastic linear operators as

(A.8) $$\begin{array}{ll} E^{*} & =\frac{4\left(q_{0}^{d}p_{0}^{m}q_{0}^{d}+2p_{0}^{d}q_{0}^{m}q_{0}^{d}\right)}{3\left(2q_{0}^{d}p_{0}^{m}p_{0}^{d}+p_{0}^{d}p_{0}^{d}q_{0}^{m}\right)}\\ & =\frac{4\left(Q^{d}P^{m}Q^{d}+2P^{d}Q^{m}Q^{d}\right)}{3\left(2Q^{d}P^{m}P^{d}+P^{d}P^{d}Q^{m}\right)} \end{array}$$

Hence, Equation (A.1) becomes

(A.9) $$\begin{array}{l} \left(2Q^{d}P^{m}P^{d}+P^{d}P^{d}Q^{m}\right)\left[F\left(t\right)+2\mathrm{\pi wR}\right]\\ \;=\frac{4\sqrt{R}}{3}\left(Q^{d}P^{m}Q^{d}+2P^{d}Q^{m}Q^{d}\right)\delta^{\frac{3}{2}}\left(t\right) \end{array}$$

To evolve the elastic solution into a viscoelastic solution, the linear operators in the viscoelastic system need to be determined. To this end, the standard solid model, shown in Figure 2(a), was used to simulate the viscoelastic behavior of the sample, since both the instantaneous and retarded elastic responses can be reflected in this model, which well describes the mechanical response of most viscoelastic bodies.

It is customary to assume that the volumetric response under the hydrostatic stress is elastic deformation; thus, it is uniquely determined by the spring in series [55]. Hence, the four linear operators for the standard solid model can be expressed as

(A.10) $$P^{d}=1+p_{1}^{d}\frac{\partial }{\partial t},Q^{d}=q_{0}^{d}+q_{1}^{d}\frac{\partial }{\partial t},P^{m}=1,Q^{m}=3K_{1}$$

where $p_{1}^{d}=\frac{\eta }{G_{1}+G_{2}},\;q_{0}^{d}=\frac{2G_{1}G_{2}}{G_{1}+G_{2}},\;q_{1}^{d}=\frac{2G_{1}\eta }{G_{1}+G_{2}},\;G_{1}=\frac{E_{1}}{2\left(1+v_{1}\right)},G_{2}=\frac{E_{2}}{2\left(1+v_{2}\right)},\;K_{1}=\frac{E_{1}}{3\left(1-2v_{1}\right)}$, *E*_1_, *E*_2_, *v*_1_, and *v*_2_ are the elastic modulus and Poisson's ratio of the two elastic components, respectively, shown in Figure 2.

Plugging Equation (A.10) into Equation (A.9), the relation between *F*(*t*) and *δ*(*t*) can be found. The functional differential equation that extends the elastic solution of indentation to viscoelastic system is obtained

(A.11) $$\begin{array}{l} \left(\sum_{i=0}^{2}A_{i}\frac{\partial^{i}}{\partial t^{i}}\right)\left[F\left(t\right)+2\mathrm{\pi wR}\right]\\ \;=\frac{4\sqrt{R}}{3}\left(\sum_{i=0}^{2}B_{i}\frac{\partial^{i}}{\partial t^{i}}\right)\delta^{\frac{3}{2}}\left(t\right) \end{array}$$

where *A*_0_ = 2*q*_0_ + 3*K*_1_, *A*_1_ = *p*_1_(3*K*_1_ + 2*q*_0_) + (3*p*_1_*K*_1_ + 2*q*_1_), *A*_2_ = *p*_1_(3*p*_1_*K*_1_ + 2*q*_1_), *B*_0_ = *q*_0_(1 + 6 *K*_1_), *B*_1_ = *q*_0_(*p*_1_ + 6*K*_1_*p*_1_) + *q*_1_(6*K*_1_ + 1), and *B*_2_ = *q*_1_(*p*_1_ + 6*K*_1_*p*_1_).

## Abbreviations

AFM: atomic force microscopy; DMT: Derjaguin-Muller-Toporov; FESEM: field emission scanning electron microscopy; JKR: Johnson-Kendall-Roberts; PVA: polyvinyl alcohol; TMV: tobacco mosaic virus.

## Competing interests

The authors declare that they have no competing interests.

## Authors' contributions

HW carried out the experiment and drafted the manuscript. XW supervised and guided the overall project and involved in drafting the manuscript. TL and BL provided the FESEM analysis on the sample. All authors read and approved the final manuscript.
